# Homoharringtonine induces apoptosis and inhibits STAT3 via IL-6/JAK1/STAT3 signal pathway in Gefitinib-resistant lung cancer cells

**DOI:** 10.1038/srep08477

**Published:** 2015-07-13

**Authors:** Wei Cao, Ying Liu, Ran Zhang, Bo Zhang, Teng Wang, Xianbing Zhu, Lin Mei, Hongbo Chen, Hongling Zhang, Pinghong Ming, Laiqiang Huang

**Affiliations:** 1 School of Life Sciences, Tsinghua University, Beijing, 100084, China; 2 The Shenzhen Key Laboratory of Gene and Antibody Therapy, State Key Laboratory of Health Science and Technology (prep), Center for Biotechnology & Biomedicine and Division of Life & Health Sciences, Graduate School at Shenzhen, Tsinghua University, Shenzhen, Guangdong, 518055, China; 3 School of Basic Medical Sciences, Hubei University of Medicine, Shiyan, 442000, Hubei, China; 4 National Laboratory of Biomacromolecules, Institute of Biophysics, Chinese Academy of Sciences, Beijing, 100101, China; 5 The Key Laboratory of Bioorganic Phosphorus Chemistry & Chemical Biology (Ministry of Education), Department of Chemistry, Tsinghua University, Beijing, 100084, China; 6 Laboratory of Zhuhai People’s Hospital, Zhuhai, Guangdong, 519000, China

## Abstract

Tyrosine kinase inhibitors (TKIs) are mostly used in non-small cell lung cancer (NSCLC) treatment. Unfortunately, treatment with Gefitinib for a period of time will result in drug resistance and cause treatment failure in clinic. Therefore, exploring novel compounds to overcome this resistance is urgently required. Here we investigated the antitumor effect of homoharringtonine (HHT), a natural compound extracted from *Cephalotaxus harringtonia*, on Gefitinib-resistant NSCLC cell lines *in vitro* and *in viv*o. NCI-H1975 cells with EGFR T790M mutation are more sensitive to HHT treatment compared with that of A549 cells with wild type EGFR. HHT inhibited cells growth, cell viability and colony formation, as well as induced cell apoptosis through mitochondria pathway. Furthermore, we explored the mechanism of HHT inhibition on NSCLC cells. Higher level of interleukin-6 (IL-6) existed in lung cancer patients and mutant EGFR and TGFβ signal requires the upregulation of IL-6 through the gp130/JAK pathway to overactive STAT3, an oncogenic protein which has been considered as a potential target for cancer therapy. HHT reversiblely inhibited IL-6-induced STAT3 Tyrosine 705 phosphorylation and reduced anti-apoptotic proteins expression. Gefitinib-resistant NSCLC xenograft tests also confirmed the antitumor effect of HHT *in vivo*. Consequently, HHT has the potential in Gefitinib-resistant NSCLC treatment.

Lung cancer is one of the mostly diagnosed cancer each year with more than one-quarter cancer patients die from lung cancer. An estimated 224,210 new cases (116,000 in men and 108,210 in women) of lung and bronchial cancer will be diagnosed in 2014, and 159,260 deaths (86,930 in men and 72,330 in women) are estimated to occur from the disease^1^. The five-year survival rate of lung cancer is 14% and 17% in men and women, respectively^2^. NSCLC accounts for approximately 80% of all lung cancer cases^3^. Some efficient agents such as PD1 and EGFR specific antibodies^4^ and reversible tyrosine kinase inhibitors (TKIs) Gefitinib^5^ and Erlotinib^6^ benefit a proportion of NSCLC patients, but treatment with Gefitinib and Erlotinib will cause drug resistance for a period of time (6~12 months) . The reasons for drug resistance are complicated and several previous studies have tried to explore the question. A secondary mutation in exon 20 of *EGFR*, T790M, and/or amplification of c-MET account for ~50% cases of Gefitinib-resistance acquisition^7^. The EGFR Threonine 790 is an important amino acid residue of inhibitor specificity in the ATP binding pocket behind the ATP binding cleft, but the substitution of Threonine 790 with Methionine increases the ATP affinity and reduce the potency of any ATP-competitive kinase inhibitor by which the T790M mutation confers drug resistance^8^. Recently, Yao *et al*. demonstrated that not only the genetic and/or epigenetic changes contribute the drug resistance, but also the activation of special tumor microenvironment do, since they found TGFβ upregulated IL-6 expression more than 10-fold and the IL-6 increased resistance to the drugs^9^. Although some irreversible EGFR TKIs, such as Afatinib^10^ or Dacomitinib^11^ were developed to overcome the drug resistance, they exhibited limited efficience. Kim *et al*. demonstrated that NSCLC cells with EGFR T790M exhibited insensitivity to Afatinib by activation of an IL-6R signaling pathway via autocrine IL-6 production. Moreover, inhibition of the JAK/STAT3 signaling potentiates the antitumor activity of Afatinib in PC9-GR xenograft models^12^. Therefore, identification and development of novel drugs which can overcome the EGFR TKI resistance is an emergency to prolong overall survival time of NSCLC patients.

Signal Transducer and Activator of Transcription (STAT) proteins are a family of transcription factors consisting of 7 members, STAT1, STAT2, STAT3, STAT4, STAT5a, STAT5b and STAT6, and they can be phosphorylated by gp130/JAKs signal^13^. JAK/STAT signal pathway malignancy may cause genomic instability, cell cycle dysregulation, and eventually formation of cancer^14^. The signal pathway is frequently constitutively activated in many cancer cell lines and tumor specimens^15^,^16^,^17^. Among the STAT family, STAT3 is an oncogenic protein and its constitutive activation is highly relevant to cancers. As a transcription factor, STAT3 regulates *Mcl1*, *Survivin*, *p21, Bcl-xl*, *Bcl-2*, *c-Myc*, *Cyclin D1*, and *VEGF* expression and therefore correlates with tumorigenesis and tumor progression. The associated processes have been experimentally indicated to have relationship with IL-6/gp130 signaling^18^. In colitis-induced cancer murine models, IL-6 plays important roles in STAT3-dependent tumorigenesis^19^,^20^. IL-6 is a multifunction cytokine and participates in acute phase inflammatory responses, glucose metabolism regulation and hypothalamic-pituitary-adrenal axis. In addition, its dysregulation causes many disease states, including many types of cancer^21^,^22^. These studies suggest that modulating IL-6 is an attractive therapeutic strategy. In a K-Ras-driven pancreatic tumor model, STAT3 activation was regulated by IL-6 and sIL-6R (a soluble form of IL-6R)^23^. Chen *et al*. also reported that IL-6 up-regulated the multidrug resistance 1 (MDR-1) gene^24^. EGFR mutations (ΔEGFR and L858R) and TGFβ can both upregulate IL-6 expression and be associated with drug resistance, which indicates that EGFR-dependent inhibition treatment maybe not sufficient for patients harboring mutant EGFR and inhibition of IL-6/gp130/JAK/STAT3 signal can further suppress growth of cancer cells and sensitize cancer cells to the antitumor drugs in NSCLC^9^,^25^,^26^,^27^.

Homoharringtonine (HHT; 4-methyl (2*R*)-2-hydroxy-2-(4-hydroxy-4-methylpentyl)butanedioate), a cephalotaxine ester, is isolated from *Cephalotaxus harringtonia* which is widely distributed in China and Japan^28^. *C. harringtonia* seeds are toxic to humans and used for Chinese traditional medicine^29^. Clinically, HHT have exhibit efficient inhibition activity against acute myelocytic leukemia (AML)^30^,^31^ and chronic myeloid leukemias (CML)^32^,^33^ alone or combined with granulocyte colony-stimulating factor, cytarabine, or interferon-α. Previous studies showed that HHT could inhibit protein synthesis by preventing aminoacyl-tRNAs binding to the peptidyl-transferase A-site cleft in the ribosome^34^. Efferth, T. *et al* found HHT was more efficient in cancer cells with wild-type p53 in a high-throughput screening assay with 55 NCI cell lines^35^. Recent studies demonstrated that the possible mechanisms of HHT in anti-myeloma may be the inhibition of AKT phosphorylation and several AKT target genes including NF-κB, XIAP, cIAP and Cyclin D1^36^ and inhibition of MCL1 protein synthesis and induction of apoptosis in chronic lymphocytic leukemia^33^. In this study, we investigated the antitumor effects and possible mechanisms of HHT on NSCLC cell lines.

## Results

### Effects of HHT on NSCLC cell lines

**Figure 1:**
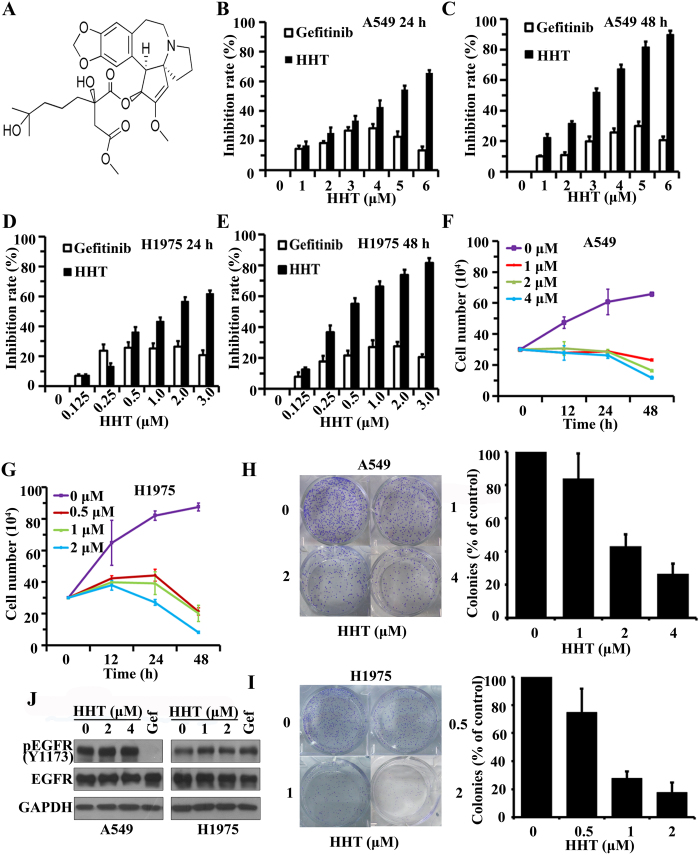
HHT inhibitory effects on NSCLC cells. (**A**): Chemical structure of HHT. (**B**–**E**): The inhibitory effects of HHT on A549 (**B** and **C**) and H1975 (**D** and **E**) cells evaluated by MTT assay. (**F** and **G**): Cell viability inhibition effect of HHT on A549 and H1975 cells analyzed by trypan blue exclusion assay. (**H** and **I**): The soft-agar colony formation assays of A549 and H1975 cells treated with HHT at indicated concentration. (**J**): A549 and H1975 cells were treated with HHT or Gefitinib for 24 h, lysed and the protein samples were analyzed by western blot with indicated antibodies. All the full-length blots are presented in Supplementary Figure 1.

In this study, we firstly investigated the cytotoxicity of HHT on human NSCLC cell lines, A549 (wild type EGFR) and NCI-H1975 (H1975, mutant EGFR with L858R and T790M), using Gefitinib as a control. By 3-(4,5-dimethylthiazol-2-yl)-2,5-diphenyltetrazolium bromide (MTT) assay, we found that HHT had moderate cytotoxicity to A549 with an IC_50_ of 3.7 μM and H1975 cells were more sensitive to HHT with an IC_50_ of 0.7 μM . We also found that HHT inhibited the cell proliferation and growth of A549 cells (Fig. 1B,C) and H1975 cells (Fig. 1D,E) in a time- and dose-dependent manner through MTT assay. By trypan blue exclusion assay, we found that HHT rapidly reduced viable A549 (Fig. 1F) and H1975 cells (Fig. 1G) in a dose- and time-dependent manner. We investigated HHT’s effect on cell colony formation activity, and the results showed that HHT significantly inhibited the clonogenic ability of A549 (Fig. 1H) and H1975 cells(Fig. 1I). These results suggested that HHT inhibited the anchorage-dependent (cell proliferation) and anchorage-independent (colony formation) growth of NSCLC cells.

The EGFR signal pathway is a crucial target in NSCLC treatment. To test the effect on EGFR of HHT, A549 and H1975 cells were treated with HHT for 24 h and lysed. By western blot, in A549 cells, unlike Gefitinib, HHT had no effect on phosphorylation downregulation of EGFR (Y1173), while in H1975 cells neither HHT nor Gefitinib failed to downregulate EGFR phosphorylation (Fig. 1J). These data indicated that HHT-induced cell growth inhibition through other mechanism differing from Gefitinib.

### HHT induces mitochondria apoptotic pathway in NSCLC cells

**Figure 2:**
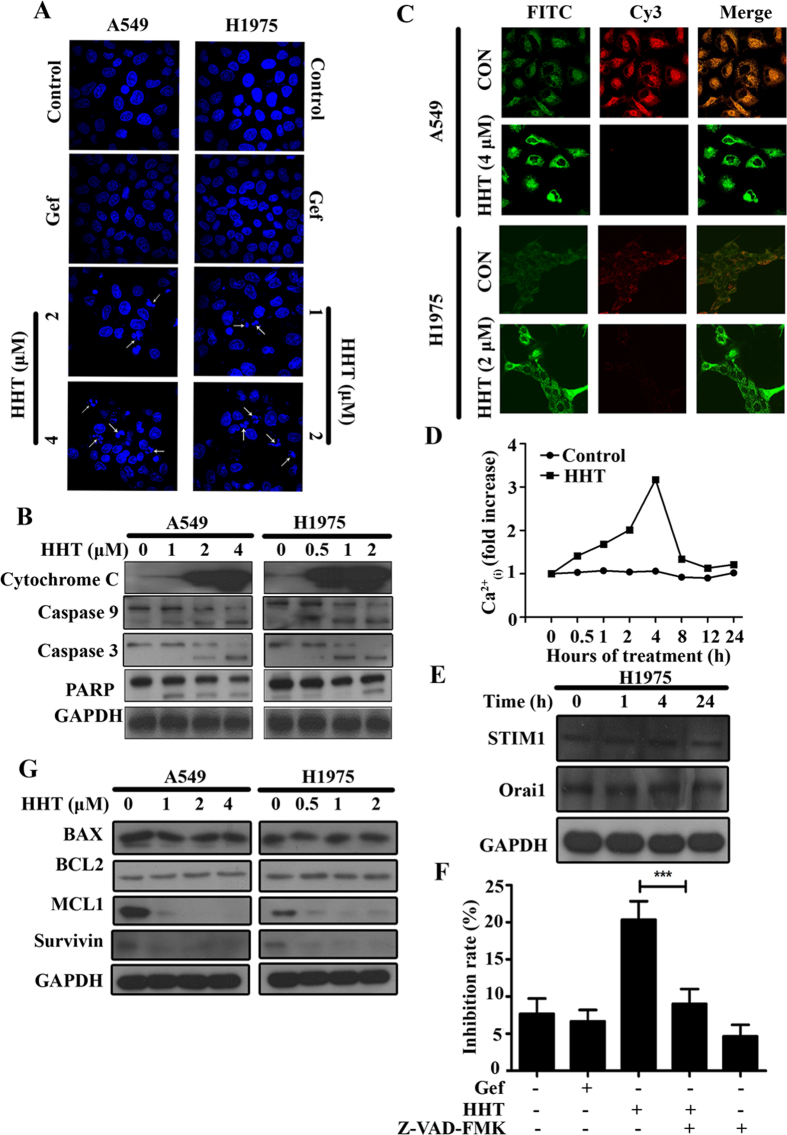
HHT induces apoptosis of NSCLC cells. (**A**): A549 and H1975 cells were treated with Gefitinib (3 mM) or HHT at indicated concentrations for 24 h and stained with Hoechst 33258 assay. (**B**): A549 and H1975 cells were treated with HHT, lysed and the protein samples were analysed by western blot with indicated antibodies. (**C**): A549 and H1975 cells were treated with HHT at indicated concentration and the mitochondrial transmembrane potential (ΔΨ) was tested by confocal microscopy (Olympus Fluoview FV-1000, Tokyo, Japan). (**D**): Ca^2+^_(i)_ was measured using Ca^2+^ indicator FLUO-4 (Invitrogen) by flow cytometry assay. (E): H1975 cells were treated with HHT for 24 h, lysed and analysed by western blot with indicated antibodies. (F): H1975 cells were pretreated with Z-VAD-FMK (20 mM) for 1 h and then treated with HHT at 2 mM for 24 h, and the inhibition rate was determined by MTT assay. The mean±SD of three independent experiments is shown. ***, *P* < 0.01. (G): A549 and H1975 cells were treated with HHT for 24 h, lysed and analysed by western blot with indicated antibodies. The blots shown are derived from multiple gels. Membrane was cut based on the molecular weight, probed with antibody of interest and band of interest is indicated with an arrow. All the full-length blots are presented in Supplementary Figure 2.

As indicated above, we tried to investigated the mechanism underlied the inhibition effect of HHT on Gefitinib-resistant NSCLC. By the optical light microscope, we found some dead A549 and H1975 cells floating in the medium treated with HHT. The cell death is reminiscent of the phenomena induced by apoptosis. Next, we tested the possibility of induction of apoptosis by HHT. Firstly, we investigated the nucleus morphological changes by Hoechst 33258 staining. As shown in Fig. 2A, we can find the nuclear condensation and fragmentation with HHT treatment which are typical changes in cell apoptosis. To identify the variation of apoptosis-related proteins, A549 and H1975 cells were treated with HHT at indicated concentration. By whole cell lysis extraction and western blot, HHT treatment resulted in a significant increase of cytochrome C release into cytoplasm and the decrease of the full length of Caspase 9, Caspase 3 and cleavage of poly(ADP-ribose) polymerase (PARP) in A549 and H1975 (Fig. 2B) cells in a dose-dependent manner. To further investigate the mitochondrial dysfunction in A549 and H1975 cells following HHT treatment, we measured mitochondrial transmembrane potential *in situ*. JC-1 accumulated in the mitochondria and formed red aggregates depending on the potential in healthy cells, and mitochondrial depolarization caused JC-1 release from mitochondria and exhibited green fluorescence. The untreated A549 and H1975 cells displayed strong red fluorescence, conversely the HHT-treated cells exhibited weak red fluorescence (Fig. 2C), suggesting the disruption of the mitochondrial transmembrane potential. Calcium (Ca^2+^) regulates many cellular functions and also participates in cell apoptosis. Mitochondrial Ca^2+^ uptake may lead to mitochondria swelling and in turn the release of mitochondrial apoptotic factors into the cytosol^37^. We examined HHT effects on intracellular calcium variation in H1975 using FLUO-4 (Invitrogen). We found there was a peak of intracellular Ca^2+^ levels at 4 h after HHT treatment and then decreased to basal level (Fig. 2D). Additionly, in lung, stromal interaction molecule 1 (STIM1)-Orai1 regulates store-operated Ca^2+^ entry (SOCE) in airway smooth muscle cells and its dysregulation correlates with pulmonary smooth muscle malignant cell proliferation. Orai1 overexpression causes the inflammatory response in cystic fibrosis and its knockdown inhibits endothelial cell migration and angiogenesis^38^. HHT had no effect on STIM1 and Orai1 during this period (Fig. 2E). To further identify the caspase dependent apoptosis induced by HHT, H1975 cells were pre-treated with a general caspase inhibitor Z-VAD-FMK (20 μM) for 1 h and then treated with HHT at 2 μM for 24 h. We found that HHT-induced apoptosis was significantly suppressed (Fig. 2F). These results indicated that HHT induced apoptosis through the mitochondrial pathway by activation of caspase cascade. Consequently, we examined the expression of several pro- and anti-apoptotic proteins and found that MCL1, a BCL2 family anti-apoptotic protein, and Survivin, other than BCL2 or BAX, decreased with HHT treatment in a concentration depend manner in A549 (Fig. 2G left panel) and H1975 cells (Fig. 2G right panel).

### HHT suppresses phosphorylation of STAT3 via inhibiting JAK/STAT3 pathway in NSCLC cells

**Figure 3:**
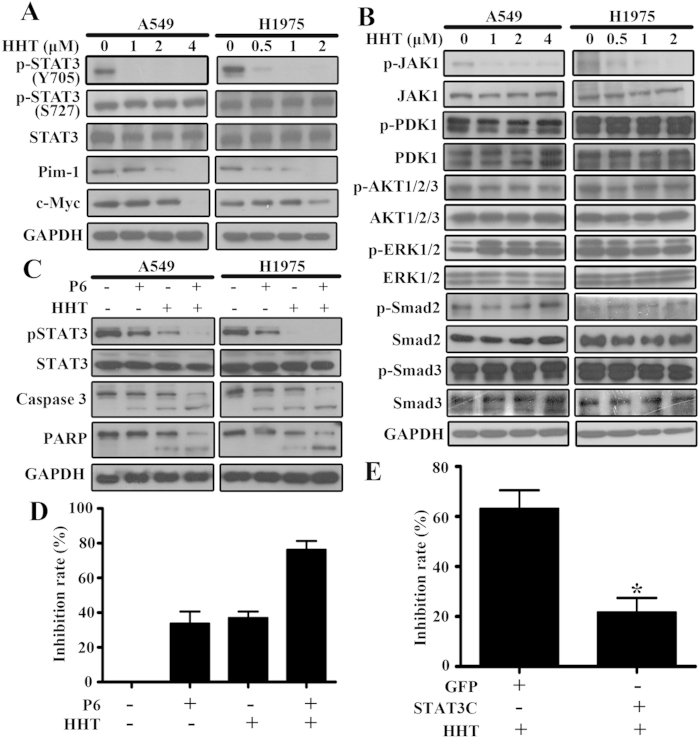
HHT supresses the phosphorylation of STAT3. (**A** and **B**): A549 and H1975 cells treated with HHT, and the STAT3 phosphorylation and its target genes (**A**) and the upstream key efftors (**B**) were examined by western blot with indicated antibodies. (**C**): With pan-JAK inhibitor P6 (1 μM) and HHT (1 μM) treatment for 12 h, A549 and H1975 cell extracts were conducted western blot with indicated antibodies. (**D**): H1975 cells were treated with P6 and HHT together or alone and conducted MTT assay. (**E**): H1975 cells transfected with STAT3C or empty vector were treated with HHT and the inhibition rate was determined by MTT assay (*P* < 0.01). The blots shown are derived from multiple gels. Membrane was cut based on the molecular weight, probed with antibody of interest and band of interest is indicated with an arrow. All the full-length blots are presented in Supplementary Figure 2.

Previous studies have shown that the *Mcl1* and *Survivin* promoters contain a common potential transcription factor STAT3 binding site^39^. Additionally, STAT3 constitutive activation promotes tumorigenesis partly through upregulation of certain antiapoptotic proteins expression including *Bcl-xL*, *Bcl2*, *Survivin*, and *Mcl1*, and has been discovered in a variety of hematological tumors and solid tumors including 22%~65% NSCLC. This aberrant constitutive activation plays malignant roles in lung cancer cell proliferation and associated with resistance to chemotherapy^40^,^41^. STAT3 is activated by a variety of cytokines and growth factors. In clinical studies, STAT3 or pSTAT3 is associated with poor prognosis^40^. We tested the expression of STAT3 and its phosphorylation states in A549 and H1975 cells treated with HHT, and showed that the STAT3 phosphorylation at Tyr705 (p-STAT3 Y705), which is related STAT3 dimerization, nucleocytoplasmic shuttling, and DNA binding^42^, was decreased, but not the phosphorylation at Ser727 (Fig. 3A). Pim-1 combined with c-Myc participates in cell transformation and protects cells from apoptosis. Additionally, Pim-1 and c-Myc are STAT3 target genes upon gp130 stimulation^43^. We also found that Pim-1 and c-Myc expression decreased upon HHT treatment in both A549 and H1975 cells (Fig. 3A). Previous studies have shown that IL-6/JAK, PI3K/AKT and RAS/MAPK/ERK signal pathways are three major upstream STATs activators, so we tested the JAK1, PDK1, AKT and ERK active status. As shown in Fig. 3B, JAK1 phosphorylation (Tyr1022/1023) was significantly decreased but the PDK1, AKT and ERK phosphorylation was not changed after HHT treatment. Additionally, Yao *et al*. demonstrated that IL-6 upregulation mediated by TGF-β signal pathway conferred NSCLC cell drug resistance in an EGFR independent manner and promoted NSCLC cell proliferation^9^. As shown in Fig. 3B, the total protein level and phosphorylation of Smad2 and Smad3 were not inhibited by HHT treatment. These results suggested that HHT inhibited the STAT3 aberrant activation through IL-6/JAK/STAT3 pathway without interference with TGFβ signal. Furthermore, we investigated the inhibition effect of combination of pan-JAK inhibitor Pyridone 6 (P6, 1 μM) and HHT (1 μM) treatment for 12 h. Extracts from A549 and H1975 were analyzed for phospho- and total STAT3, Caspase 3 and PARP by western blot. As shown in Fig. 3C, the phosphorylated STAT3 was further inhibited by P6 and HHT combination with no variation of total STAT3. The Caspase 3 was also activated by the combination. Additionally, the cell inhibition rates were also augmented by the combination assayed by MTT assay in H1975 cells (Fig. 3D). To further investigate the essential role of STAT3 inhibition in HHT-induced cell death, H1975 cells transfected with EF.STAT3C.Ubc.GFP were treated with 2 μM HHT for 24 h and the cell inhibition rate was determined by MTT assay. As shown in Fig. 3E, the overexpression of constitutively active mutant of STAT3 (STAT3C) exhibited resistance to HHT treatment and the inhibition rate significantly decreased compared with control group.

### HHT inhibits IL-6-induced STAT3 phosphorylation in a dose- and time-dependent manner

**Figure 4:**
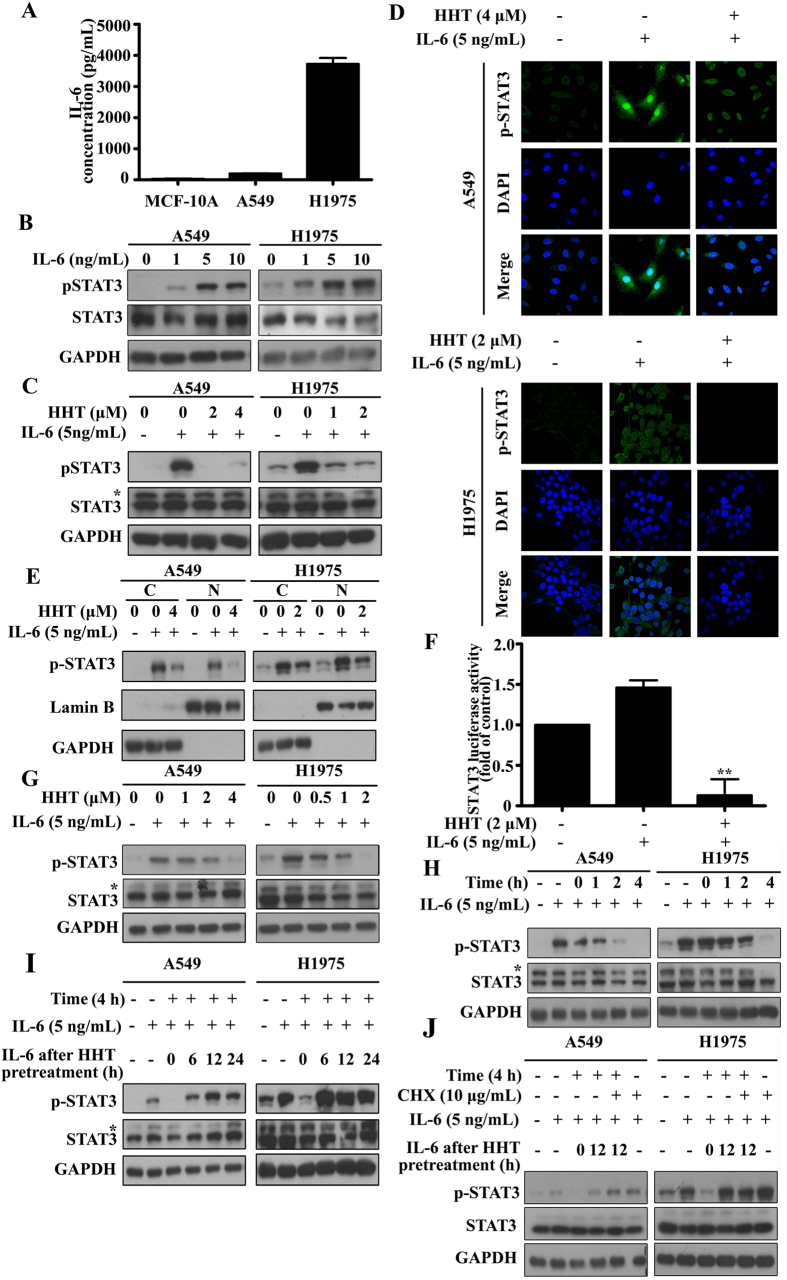
HHT inhibits IL-6-induced STAT3 phosphorylation a dose- and time-dependent manner. (**A**): IL-6 production in MCF-10A, A549 and H1975 cells measured by ELISA. (**B**): Cells were starved and treated with different concentration IL-6. Protein samples were detected by western blot. (**C**–**E**): Cells were starved and pretreated with PBS or HHT for 4 h followed by IL-6 treatment. Protein samples were detected by western blot (**C**), the distribution variation of phosphorylated STAT3 (Y705) were examined by immunofluorescence (**D**) and nuclear (N) and cytoplasmic (**C**) isolation assay (**E**). (**F**): pSTAT3-TA-luc plasmids were transfected into H1975 cells followed by treatment with HHT for 4 h. Then H1975 cells were treated with IL-6 for another 20 h. Firefly luciferase activities were assayed. (**G**): Cells were starved and pretreated with HHT at different concentrations followed by IL-6 stimulation. Protein samples were examined by western blot. (**H**): Cells were starved and treated with 4 μM (in A549 cells) or 2 μM (in H1975 cells) for indicated time points (0 h–4 h). After HHT pretreatment, cells were treated with 5 ng/mL IL-6 for 30 min. Protein samples were detected by western blot. (**I**): Cells were starved and pretreated with 4 μM (in A549 cells) or 2 μM (in H1975 cells) HHT for 4 h. Discard the HHT-containing medium and add fresh medium without HHT. After indicated incubation times (0 h–24 h), cells were treated with 5 ng/mL IL-6 for 30 min. Protein samples were detected by western blot. (**J**): Cells were starved and then pretreated with 4 μM (in A549 cells) or 2 μM (in H1975 cells) for 4 h. Discard the HHT-containing medium and add fresh medium containing protein synthesis inhibitor CHX (10 μg/mL) without HHT. After 12 h, cells were stimulated with 5 ng/mL IL-6 for 30 min. Protein samples were detected by western blot. The blots shown are derived from multiple gels. Membrane was cut based on the molecular weight, probed with antibody of interest and band of interest is indicated with an arrow. All the full-length blots are presented in Supplementary Figure 3.

Apart from EGFR, STAT3 can be activated via some inflammatory cytokines as well as growth factors. Previous studies have shown that EGFR mutation increased IL-6 expression and therefore activated STAT3 via IL-6/gp130/JAK1 signal pathway^25^,^44^ and JAK1 was the critical JAK receptor kinase in IL-6/gp130/JAK/STAT3 signal pathway in lung cancer cells^27^. *In vivo* study also suggested IL-6 blockage inhibited STAT3 activation and therefore repressed H1650 xenografts cell growth^27^. We found that IL-6 expression in a very low level in non-transformed breast epithelial MCF-10A and wild type EGFR harboring A549 cells, but the IL-6 production (3723 pg/mL) was significantly elevated in H1975 cells harboring mutant EGFR (Fig. 4A), which was consistant with the previous results^25^. To examine if HHT can block IL-6-induced STAT3 activation, we firstly validated the IL-6 activation effect on STAT3 activation. A549 and H1975 cells were cultured in DMEM and RPMI-1640 medium for 12 h, and then were starved in serum-free mudium for another 12 h or more time. The cells were treated with IL-6 at different concentration for 30 min. As shown in Fig. 4B, IL-6 can activate STAT3 by phosphorylation in a dose-dependent manner, and we choose 5 ng/mL IL-6 for subsequent experiment. Next, with the same culture conditions, after starvation, A549 cells were pretreated with 2 μM or 4 μM HHT and H1975 cells were pretreated with 1 μM or 2 μM HHT for 4 h followed by 5 ng/mL of IL-6 stimulation for 30 min. Fig. 4C showed that IL-6 induced STAT3 phosphorylation but the induction was repressed by HHT-pretreated cells. Meanwhile, the total STAT3 expression level was not altered with IL-6 or HHT treatment. Previous studies have shown that STAT3 phosphorylation was associated with translocation between nuclear and cytoplasm^42^. To investigate whether HHT can block this IL-6-induced translocation, cells were pretreated with indicated concentration HHT for 4 h and then incubated the cells with 5 ng/mL IL-6 for 30 min. Cells were fixed with 4% paraformaldehyde diluted in 1× PBS and immunofluorescence-stained with anti-phosphorylated STAT3 (Y705) primary antibody and FITC-conjugated secondary antibody. The nucleuses were stained with 5 μg/mL DAPI. As shown in Fig. 4C, STAT3 was activated and translocated into nucleus with IL-6 stimulation, but there were weaker STAT3 activation and nucleus translocation with HHT pretreatment. To further confirm the HHT-induced inhibition of STAT3 translocation on IL-6 stimulation, A549 and H1975 cells were treated as above and the nuclear and cytoplasmic fractionations were isolated and analyzed by western blot. As shown in Fig. 4E, HHT pretreated cells showed relatively weaker phosphorylated STAT3 in the nucleus. Additionally, HHT also inhibited STAT3 transcription activity in H1975 cells, which further confirmed HHT inhibited the nuclear translocation of p-STAT3 (Fig. 4F). The above results suggested that HHT pretreatment could block IL-6-induced STAT3 phosphorylation and nuclear translocation.

To further investigate if the HHT repression of IL-6-induced STAT3 phosphorylation is in a dose-dependent manner, we pretreated A549 and H1975 cells with different concentrations of HHT (0–4 μM) for 4 h following treatment with 5 ng/mL of IL-6 for 30 min. As shown in Fig. 4G, the HHT-induced inhibition was dose dependence. 4 μM HHT in A549 cells and 2 μM in H1975 cells would dramaticly reduce the STAT3 phosphorylation with no alternation of STAT3 expression level.

To examine if the HHT inhibition of IL-6-induced STAT3 phosphorylation is time dependence, we pretreated A549 and H1975 cells with 4 μM and 2 μM HHT, respectively, for different time points followed by 5 ng/mL of IL-6 for 30 min. Fig. 4H suggested that HHT inhibited IL-6-induced STAT3 phosphorylation was time depdence. 4 h HHT pretreatment would reduce the STAT3 phosphorylation and STAT3 expression level did not change.

To explore if the HHT inhibition on IL-6-induced STAT3 activation could be reversible, after serum starvation for 12 h, A549 and H1975 cells were pretreated with HHT for 4 h. Following the 4 h treatment, the HHT-containing medium was changed for HHT-free medium for further culture for indicated time points, and then the cells were treated with 5 ng/mL IL-6 for 30 min. As shown in Fig. 4I, after 6 h with HHT treatment, STAT3 phosphorylation in A549 and H1975 cells almost recovered. The results suggested that the HHT inhibition on IL-6-induced STAT3 phosphorylation in NSCLC was reversible. Cells were further blocked in protein biosynthesis using 10 μg/mL of cycloheximide (CHX) and repeat the above treatment in Fig. 4H to explore if IL-6 could induce STAT3 phosphorylation 6 h after HHT treatment. As seen in Fig. 4J, we found the recovery of STAT3 phosphorylation in CHX-treated cells. The results suggested that the STAT3 phosphorylation recovery was not derived from the new synthesized STAT3 protein.

**Figure 5:**
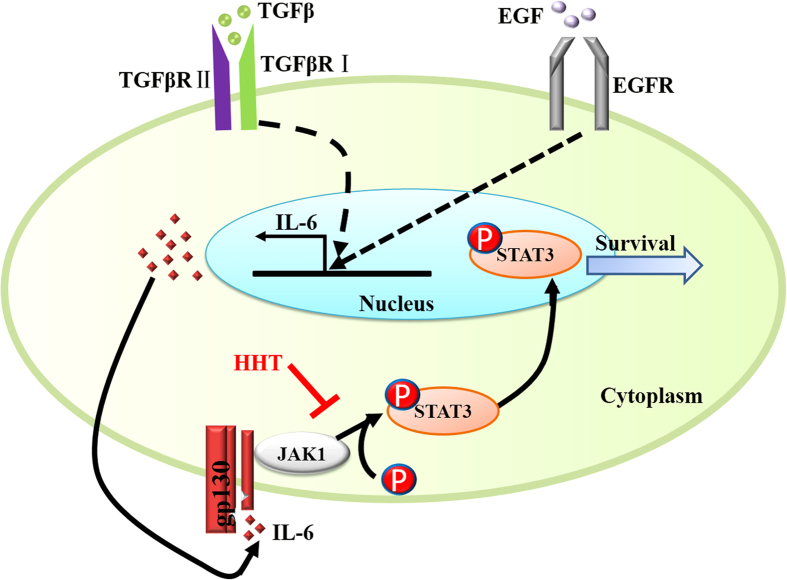
Diagram of HHT blockage possible mechanism in NSCLC cells.

From the above, we can draw a conclusion that HHT inhibited NSCLC growth and cell viability and induced cancer cell apoptosis mainly through the inhibition of IL-6/JAK/STAT3 signal pathway (Fig. 5).

### HHT exerts synergistic effect combining with Docetaxel in NSCLC cell

**Figure 6:**
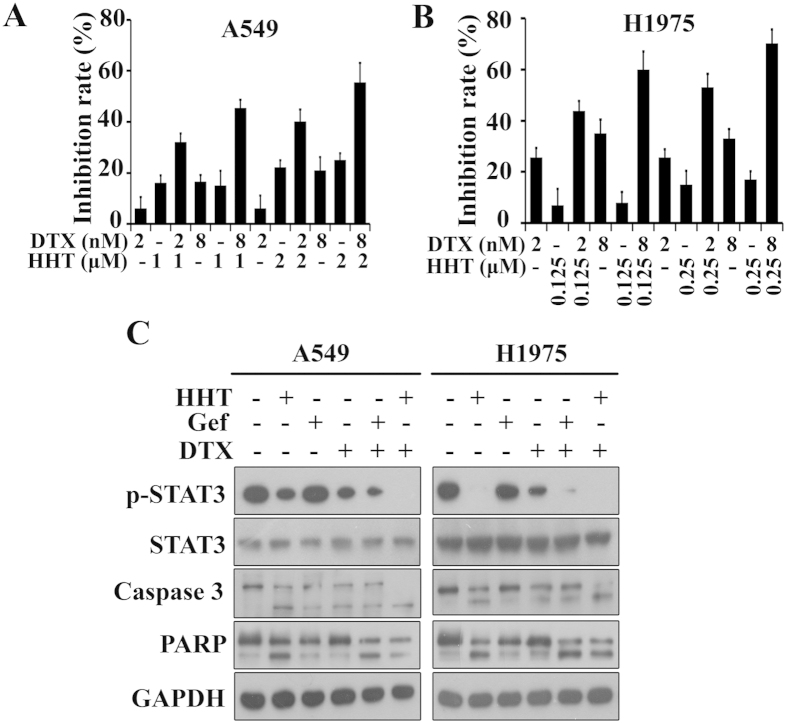
HHT exerts synergistic effect combining with docetaxel. (**A** and **B**): A549 (**A**) and H1975 (**B**) cells were treated for 24 h with DTX and/or HHT, and then assessed by MTT assay. (**C**): A549 and H1975 cells were treated with HHT (2 μM or 0.25 μM), Gefitinib (2 mM), DTX (8 nM) alone or together for 24 h. The treated cells were collected, lysed and assessed by western blot with indicated antibodies. The blots shown are derived from multiple gels. Membrane was cut based on the molecular weight, probed with antibody of interest and band of interest is indicated with an arrow. All the full-length blots are presented in Supplementary Figure 4.

In order to improve therapeutic efficacy and reduce the drug toxicity to patients or/and non-target tissues, one or more other drugs usually are used in clinic. Furthermore, an optimal combined treatment may decrease or postpone the drug resistance^45^. Docetaxel (DTX) can bind to microtubules, cause cell-cycle arrest and apoptosis and therefore is approved to cure a certain of tumors including NSCLC^46^. We examined whether HHT and DTX combination had the synergistic effect in NSCLC treatment. By MTT assay, HHT-induced cell proliferation inhibition was significantly elevated by DTX at 2 nM to 8 nM (Fig. 6A,B). We also analyzed the CI value by the formula^47^ assisted with CalcuSyn software (Version 2.1) and found that the CI values were less than 1 (Table 1), which indicated that HHT and DTX played synergistic effect in Gefitinib-resistant NSCLC cells. By western blot, we further confirmed the synergistic effect and found that HHT and DTX combination resulted in elevated levels of Caspase 3 activation, cleavage of PARP and decreased level of STAT3 phosphorylation (Fig. 6C).

### HHT exhibits antitumor effect on NSCLC xenograft tumor *in vivo*

**Figure 7:**
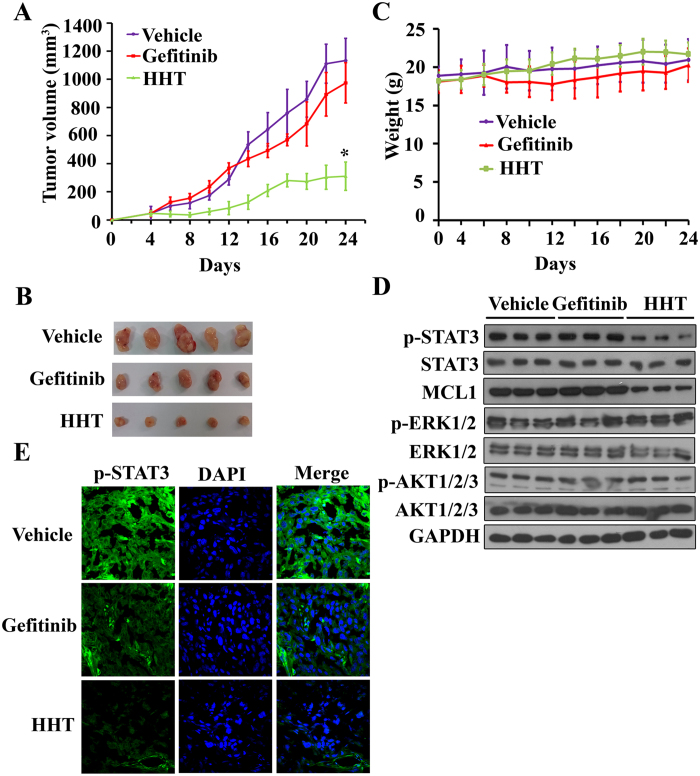
*In vivo* therapeutic efficiency of HHT on mice xenograft bearing human Gefitinib-resistant H1975 cells. (**A**): Murine models were treated with Gefitinib or HHT and the tumor volumes were calculated every two days. (**B**): Images of xenograft tumors obtained from mice with different treatment after 3 weeks. (**C**) HHT treatment did not affect the murine model body weight. (**D**): Phosphorylated STAT3(Y705), MCL1 expression level, phosphorylated AKT and ERK of tumor sample lysates were analyzed by western blot with indicated antibodies. Vehicle (Line 1–3), Gefitinib (Line 4–6) and HHT (Line 7–9). The blots shown are derived from multiple gels. Membrane was cut based on the molecular weight, probed with antibody of interest and band of interest is indicated with an arrow. All the full-length blots are presented in Supplementary Figure 5. (**E**): Phosphorylated STAT3(Y705) expression was examined by tumor immunofluorescence staining.

To access the anti-tumor effect on NSCLC *in vivo*, we subcutaneously inoculated 2.5 × 10^6^ H1975 cells in 100 μL serum-free RPMI-1640 medium into the right flank of nude mice to generate xenografted murine models. When the tumors grow to a measurable size, each group (10 mice for each random allocation) were administrated with vehicle control, Gefitinib (30 mg/kg) and HHT (10 mg/kg) 5 times per week for 3 weeks. Tumor bearing mice were humanely killed when their tumors reached 2 cm indiameter or when paralysis or major compromise in their quality of life occurred. To our expections, we found that HHT efficiently repressed tumor growth compared to vehicle control or Gefitinib (*P* < 0.05) (Fig. 7A,B). Additionally, HHT treatment did not reduce the mice body weight, which suggested that HHT had no apparent side effect (Fig. 7C). All the mice were euthanized, the tumors were isolated and imaged and the tumor sample cells were harvested to extract protein for determination if HHT inhibited STAT3 phosphorylation via western blot. As seen in Fig. 7D, the level of STAT3 phosphorylation and MCL1 from HHT treatment group was significantly decreased compared to vehicle control or Gefitinib treatment. Meanwhile, consistant with the results in the above, AKT1/2/3 and ERK1/2 phosphorylation was not inhibited with HHT treatment.To further examine the STAT3 phosphorylation in the xenograft tumor samples with different treatments, the tumor samples were frozen and cutted into 10 μm sections for fluorescent immunohistochemistry. Figure 7E showed that HHT treatment inhibited STAT3 phosphorylation compared to vehicle control or Gefitinib treatment. These results suggested that HHT had immense potential for Gefitinib-resistant NSCLC therapy.

## Discussion

Lung cancer is the major cancer type all over the world^1^ and several efficient antibodies and drugs are developed for treatment^4^,^5^,^6^. As an effective target, EGFR locates on the cell surface and is overactivated in ≥50% NSCLC patients, which leads to uncontrolled activation of anti-apoptotic signal pathway and uncontrolled cell proliferation. Gefitinib is the first generation EGFR reversible inhibitor. Although many patients are sensitive to Gefitinib in the early treatment, there will be resistance to it due to EGFR second mutant and IL-6 overexpression^12^,^25^. To overcome such resistance, exploring novel compound seems extremely urgent. Compounds from natural source constitute an indispensable candidate drug library for pharmacotherapy. In this study, we studied the antitumor effect of HHT, a cephalotaxine esterisolated from *C. harringtonia*, on the NSCLC cells growth inhibition, colony formation repression, apoptosis induction, as well as xenograft tumor suppression.

Induction of cell apoptosis is an efficient strategy in cancer therapy. In this present study, we firstly showed that HHT inhibited Gefitinib-resistant NSCLC A549 and H1975 cells proliferation (Fig. 1B–D), cell viability (Fig. 1E,F) and soft-agar colony formation (Fig. 1G,H). Interestingly, HHT has no effects on EGFR phosphorylation which differs from that of Gefitinib (Fig. 1J). In addition, we found that the change in cellular morphology and nucleus condensation, which were typical characters of apoptosis. Therefore, HHT might have the ability of induction of cell apoptosis. The mitochondria function dysregulation is one of apoptosis incentive. We tested apoptosis-related proteins and found that HHT treatment induced the release of cytochrome C from mitochondria to cytoplasm, the activation of Casapse 9 and Caspase 3 and the cleavage of PARP (Fig. 2B). In the mitochondria pathway of apoptosis, mitochondrial transmembrane potential disruption is a landmark event in the early stage of the induction of apoptosis. Therefore, we also found the disruption of mitochondrial transmembrane potential (Fig. 2C) and Ca^2+^ level fluctuation at different time points(Fig. 2D). Consequently, HHT could induce mitochondria apoptotic pathway in NSCLC cells.

STAT3 is a key effector of JAK/STAT downstream signal pathway. Many cytokines and growth factors, such as EGF, IL-5, IL-6, HGF, LIF and so on, bind to their corresponding receptors respectively and phosphorylate tyrosine 705 of STAT3 through receptor-associated kinases^40^. As a transcription factor, STAT3 regulates a variety of genes expression following the cell stimuli and consequently greatly impacts many cellular behaviors such as growth, migration, apoptosis and autophagy^40^,^42^. STAT3 persistent phosphorylation has been found in 22%~65% NSCLC and this aberrant constitutive activation is correlated with lung cancer cell proliferation, resistance to chemotherapy and poor prognosis^40^,^41^. In the present work, we tested and found that HHT treatment could inhibit the STAT3 Y705 phosphorylation and the translocation to cellular nuleus to regulate the anti-apoptotic proteins expressions, such as MCL1 and Survivin (Fig. 2A–F). Previous study in multiple myeloma suggested that HHT inhibited AKT phosphorylation and induced cell death^36^. However, HHT treatment in A549 and H1975 inhibited JAK1 phosphorylation but not AKT, ERK, Smad2 or Smad3 phosphorylation (Fig. 3B), which might result from different cell contexts and microenvironment. Moreover, pan-JAK inhibitor P6 enhanced the effects of HHT on apoptosis inductions (Fig. 3C,D). The STAT3C overexpression attenuated cell death rate which further comfirmed the STAT3 essential role in HHT-induced cell apoptosis (Fig. 3E).

Previous studies have demonstrated that higher levels of IL-6 existed in advanced and metastatic cancer patients in their blood including the lung cancer patients. Scott *et al*. reported that patients with NSCLC have increased levels of serum IL-6 and C-reactive protein that correlate with decreased survival and weight lose^48^. Gao *et al*. found that 50% specimens contained nuclear pSTAT3 through immunohistochemical analysis of tissue microarrays (TMAs) of primary lung adenocarcinomas (92 tumor specimens) and there existed a strong correlation between mutant EGFRs and pSTAT3. Since cell lines harboring mutant EGFRs (in-frame deletions in exon 19 and L858R) also produce high IL-6 levels and the STAT3 was activated through the IL-6/gp130/JAK pathway which could be inhibited with P6 or gp130 blockade, they performed immunohistochemical analysis of TMAs and found that pSTAT3 levels correlate positively with IL-6 expression. This finding suggested the strong correlation between EGFR mutations and IL-6 expression in NSCLC patients^25^. Previous researches also showed that IL-6 played an important role in regulation of the immune response and cell proliferation and neutralizing IL-6 with anti-IL-6 antibodies affected cell proliferation. In NSCLC cells which harbor mutant EGFR overactivate STAT3, but this activation is not direct driven by EGFR since this process requires the upregulation of the IL-6 via the gp130/JAK pathway^12^,^27^,^40^,^44^,^49^. Kim *et al*. demonstrated that NSCLC cells with EGFR T790M exhibited insensitivity to Afatinib (irreversible EGFR kinase inhibitor) by activation of an IL-6R signaling pathway via autocrine IL-6 production. Moreover, inhibition of the JAK/STAT3 signaling potentiates the antitumor activity of Afatinib in PC9-GR xenograft models^12^. Furthermore, several studies showed that the T790M mutation may also enhance the catalytic activity of EGFR and confer a growth advantage^50^,^51^. Yao *et al*. demonstrated that in Erlotinib and Gefitinib resistant cells lacking of any known mutations that confer resistance to cells, TGFβ upregulated IL-6 expression more than 10-fold and the IL-6 increased resistance to the drugs. NSCLC samples isolated from Tarceva-naïve patients analysis showed that a subpopulation of cells had high levels of TGFβ and IL-6^9^. The above investigations show that while EGFR mutations have a strong correlation with IL-6 expression in a portion of lung cancer patients, there are many other known (TGFβ, for example) and unkonwn factors contributing to IL-6 expression upregulation. Consequently, treatments based on EGFR inhibition may not be sufficient for the effective treatment of lung-cancer patients with mutant EGFRs and it is necessary to inhibit IL-6/gp130/JAK pathway and further repress STAT3 activity in NSCLC treatment strategy. In this study, we confirmed that IL-6 could phosphorylate STAT3 (Y705) (Fig. 4B) but this induction was inhibited by HHT treatment (Fig. 4C) and the STAT3 nucleus translocation and transcription activity were simultaneously repressed (Fig. 4D–F). There was no alteration of STAT3 expression with HHT treatment (Fig. 4B,C). We also found in the blockade of IL-6-induced STAT3 phosphorylation, the effect of HHT was in a dose- and time-dependent manner. Cells were pretreated with 1 μM or 0.5 μM HHT in A549 or H1975 cells respectively would inhibit 5 ng/mL IL-6-induced STAT3 activation (Fig. 4G,H) and the STAT3 activation was inhibited by HHT 4 h pretreatment(Fig. 4G). After 6 h with HHT treatment, IL-6-induced STAT3 phosphorylation in A549 and H1975 cells almost recovered which indicated that the HHT inhibition on STAT3 phosphorylation in lung cancer was reversible (Fig. 4I) and the recovery was not due to new synthesized STAT3 protein by CHX-treatment experiment (Fig. 4J).

As a second-line drug of non-small-cell lung cancer, DTX was clinically applied approved by the US Food and Drug Administration (FDA)^52^. Additionally, previous study also showed that both polymerizing and depolymerizing antimicrotubule agents have apoptosis-inducing activity^53^. We combined DTX and HHT to investigate the possible synergistic effect and the results showed that DTX at 2 nM to 8 nM (Fig. 6A,B) could significantly elevate HHT anti-tumor efficiency and the CI < 1 calculated by CalcuSyn software (Version 2.1). Moreover, the full length of Caspase 3 was significantly reduced and PARP was cleaved. Additionally, the phosphorylation of STAT3 Y705 was also significantly inhibited by HHT and DTX combination treatment. As a microtubule depolymerization inhibitor, DTX hyper-stabilizes cytoskeleton and limits cells flexibility and consequently induces cell apoptosis^54^. Intrestingly, the two mechanism-different drugs synergistically inhibited the cell growth and the combination may have clinic potential in NSCLC treatment.

We subcutaneously injected H1975 cells into nude mice and treated the animals with vehicle control, Gefitinib and HHT. After 3-weeks-treatment, HHT significantly repressed tumor growth, but Gefitinib did not exhibit comparative effect (Fig. 7A–C). We euthanized the tumor-bearing mice, collected the tumors, extracted the proteins and found that HHT downregulated the STAT3 phosphorylation (Fig. 7D). The fluorescent immunohistochemistry also confirmed the inhibition (Fig. 7E). Consequently, the above data suggested that HHT inhibited IL-6/JAK/STAT3 signal pathway and therefore induced cell apoptosis so that repressed cancer cell growth and viability. Taken together, HHT is a novel potential natural compound for patients with NSCLC in a EGFR-independent manner.

## Methods

### Ethics statement

All animal studies were conducted according to protocols approved by the Tsinghua University Animal Care and Use Committee, complying with the rules of REGULATIONS FOR THE ADMINISTRATION OF AFFAIRS CONCERNINGEX-PERIMENTAL ANIMALS (Approved by the State Council of China). The methods were carried out in accordance with the approved guidelines.

### Reagents

Homoharringtonine (HHT) with a purity of ≥98% was purchased from Aladdin Industrial Inc. (Shanghai, PR China). HHT was dissolved in phosphate buffered salineat (PBS) at a stock solution of 2.5 mM and kept at −20 °C. Docetaxel was provided by Beijing InnoChem Science & Technology Co., Ltd (Beijing, PR China). The 3-(4,5-dimethylthiahiazozy1)-3,5-di-phenytetrazoliumromide (MTT), Trypan Blue and Hoechst 33258 were purchased from Sigma-Aldrich. Interleukin 6 (IL-6) with a purity of ≥98% was purchased from EMD Millipore and dissolved in PBS at a stock solution of 100 μg/mL and stored at −20 °C. Cycloheximide (CHX) with a purity of ≥98% was also purchased from EMD Millipore and dissolved in ethanol at a stock solution of 10 mg/mL and stored at −20 °C. The JC-1 Apoptosis Detection Kit was obtained from KeyGEN Biotech Co., Ltd. (Nanjing, China). All other chemicals of the highest quality were commercially available and used as received.

### Antibodies

The antibodies used in this study were as follows: anti-PDK1, anti-phospho-PDK1 (Ser241) (GeneTex); anti-phospho-AKT1/2/3 (Ser 473) and AKT1/2/3, anti-ERK1/2 and anti-phospho-ERK1/2 (Thr202/Tyr204) (Santa Cruz Biotechnology); anti-Caspase 3 (3G2), anti-Caspase 9, anti-PARP, anti-STAT3, anti-phospho-STAT3 (Y705) and (Ser727) (Epitomics); anti-JAK1, anti-phospho-JAK1 (Y1022/1023), anti-Smad2, anti-phospho-Smad2 (Ser467), anti-Smad3, anti-phospho-Smad3 (Ser423/425) (Cell signaling); anti-GAPDH (Sangon Biotech., AB10016); anti-rabbit or anti-mouse HRP-conjugated secondary antibody (Pierce). Detection was performed by using a Chemiluminescent Western detection kit (Cell Signaling).

### Cell culture

Human NSCLC cell lines MCF-10A, A549 and H1975 were obtained from American Type Culture Collection (ATCC). MCF-10A cells were cultured in 10A medium as previously described^55^. A549 cells were cultured in Dulbecco modified Eagle medium (DMEM). H1975 cells were cultured in RPMI 1640 medium. DMEM and RPMI 1640 medium were supplemented with 10% fetal bovine serum (FBS) (Hyclone), 100 U/mL penicillin, 100 μg/mL streptomycin and cultured in a humidified atmosphere with 5% CO_2_ at 37 °C. EF.STAT3C.Ubc.GFP (lentiviral expression of constitutively active STAT3, A661C and N663C) was a gift from Linzhao Cheng^56^ (Addgene plasmid # 24983) and its empty vector FUGW was a gift from David Baltimore^57^ (Addgene plasmid # 14883). H1975 cells were transfected with lentiviruses expressing these plasmids and sorted for a high green fluorescent population for further experiment.

### Cytotoxic assay and cell viability

Cells were seeded into 96-well plate and precultured for 24 h, then treated with HHT for 24 h or 48 h. Cell cytotoxicity was determined by MTT assay. The absorbance was measured at 570 nm by Varioskan™ Flash Multimode Reader (Thermo Fisher Scientific, USA), and the cell death rate was calculated as followed: Cell death (%) = (average *A*_570_ of the control group - average *A*_570_ of the experimental group)/(average *A*_570_ of the control group - average *A*_570_ of the blank group)^58^.

Cell viability was estimated by trypan blue dye exclusion assay. The cells which exclude the dye are viable. Place 0.5 mL of a suitable cell suspension (dilute cells in complete medium without serum to 1 × 10^6^ cells per mL) following adding 0.1 mL of 0.4% trypan blue dye and mixing thoroughly, and then incubate at room temperature for 3 min and load into a hemacytometer to count cells in three separate fields (nonviable, deep blue cells as well as viable, clear cells). The cell viability rate was calculated as followed: Cell viability (%) = (number viable cells/number total cells)×100%^59^. After staining with Hoechst 33258 at 10 mg/mL for 10 min, cell death was observed by a fluorescence microscope (Leica).

### Soft-agar colony formation assay

A549 and H1975 cells (1000 cells per plate) were suspended in 1 mL of DMEM or RPMI 1640 containing 0.3% low-melting-point agarose (Amresco, USA), 10% FBS and indicated concentration of HHT, andplated on a bottom layer containing 0.6% agarose and 10% FBS in 6-well plate intriplicate. After 2 weeks, plates were stained with 0.5 mL of 0.005% crystal violet for more than 1 h and the colonies were counted under light microscope^60^.

### Ca^2+^_(i)_ measurement

H1975 cells were treated with HHT and harvested with cell dispersant (Sigma Aldrich) followed by incubation with Ca^2+^ indicator FLUO-4 (Invitrogen) for 60 s. Then flow cytometry assay was performed for detection for intracellular Ca^2+^ levels (Ca^2+^_(i)_) using the FL1 channel.

### Solid phase sandwich ELISA for IL-6

When cells were cultured to approximately 50%-85% confluence, the medium was aspirated followed by washing with PBS twice, and fresh medium was added. After 12 h, the conditioned medium was collected and centrifuged for 15 minutes at 3,000 rpm to remove the dead cells. The collected media were stored at −80 °C. The culture cells were harvested by trypsinization and counted for normalization^12^. Analysis for IL-6 was conducted with Human IL-6 Quantikine ELISA Kit (R&D Systems) according to the manufacturer’s instructions.

### Isolation of cytoplasmic fractionation from mitochondria

To detecte the Cytochrome C released from the mitochondria caused by the possible cell apoptosis, cytoplasmic fractions were isolated from A549 and H1975 cells after incubation with cytosol lysis buffer (0.01% digitonin, 2 mM EDTA, 1 mM PMSF, protease inhibitors cocktails in 1×PBS) for 3 min at room temperature. Centrifugate for 2 min at 16,000 ×*g*, aliquots of the supernatant (cytoplasmic fractions) were collected and analyzed by western blot^59^.

### Isolation of nuclear and cytoplasmic fractionations

The treated cells were fistly washed and scraped in cold PBS (1 mL per 100 mm dishes) and then transferred into an Eppendorf tube. Collected the cells by centrifugation (2,000 rpm for 4 min at 4 °C) and resuspend and incubated the cells in 400 μL Buffer A (10 mM HEPES, pH 7.9, 10 mM KCl, 0.1 mM EDTA, 1 mM DTT and protease inhibitors) on ice for 10 min, and then add 10 μL of 10% NP-40 to the cell for further lysis. Collected the supernatant by centrifugation (6,000 rpm at 4 °C for 4 min) to obtain the cytoplasmic fractionation. The pellet was resuspended in 50 μL Bufffer B (20 mM HEPES, pH 7.9, 0.4 M NaCl, 1 mM EDTA, 1 mM DTT and protease inhibitors) and incubated on ice for 15 min to dissolve the nuclear proteins. The isolation effect was assessed by western blot using Lamin B and GAPDH for control, respectively.

### JC-1 apoptosis detection

For detection of the variation of mitochondrial transmembrane potential (ΔΨ) between healthy and apoptotic cells, A549 and H1975 cells were treated with HHT and detected by JC-1 Apoptosis Detection Kit following the manufacturer’s instructions. Briefly, after treatment by indicated concentration of HHT for 24 h, the cells were gentlely washed with PBS twice followed by adding 100 μL JC-1 work solution to the glass slides and continued to culture in a humidified atmosphere with 5% CO_2_ at 37 °C. After 20 min, washed the glass slides with 1× incubation buffer and detected the cells by fluorescence microscope.

### Immunofluorescence staining

A549 and H1975 cells were treated in the presence or absence of HHT with indicated concentration for 24 h. Cells were fixed by 4% paraformaldehyde and penetrated by 0.1% Triton X-100. A primary antibody against pSTAT3 (Y705) was added at a dilution of 1:50 and incubated with cells at 4 °C overnight. A FITC conjugated goat anti-rabbit IgG antibody was used as the secondary antibody. Meanwhile, the cell nucleus were stained by DAPI. The glass slides were then observed using an Olympus laser scanning confocal microscope with an imaging software (Olympus Fluoview FV-1000, Tokyo, Japan).

### STAT3 luciferase reporter assay

H1975 cells were seeded in 12-well plates. When grew to a confluency of 60%–70%, cells were transfected with the pSTAT3-TA-luc plasmids (D2259, Beyotime Institute of Biotechnology) using the Lipofectamine 2000 (Invitrogen) following the manufacturer’s instruction. After transfection for 4 h, cells were changed for fresh mudium and treated with HHT for 4 h followed by IL-6 treatment for another 20 h. Firefly luciferase activities were assayed using the Luciferase Assay System (Promega) according to the manufacturer’s instructions.

### Drug combination assay

Drug combination is widely used in cancer treatment to achieve synergistic therapeutic effect. To estimate the effect of HHT and Docetaxel (DTX) combination, the combination index (CI) was calculated by the Chou-Talalay equation^47^. A549 and H1975 cells were seeded in 96-well plates. Drugs were added alone or together at indicated concentration. The inhibition effect was measured by MTT assay as mentioned above. The formula of CI = (D)HHT/(Dx)HHT + (D)DTX/(Dx)DTX ((D)HHT and (D)DTX: the doses of compounds HHT and DTX, respectively, necessary to produce the same effect in combination. Dx: the dose of one compound alone required to produce an effect). With this formula and assistance of CalcuSyn software (Version 2.1), the combined effects of the two compounds can be assessed as followes: CI < 1 indicates synergism; CI = 1 indicates additive effect; and CI > 1 indicates antagonism.

### Human NSCLC xenograft experiments

Equal amounts of female and male nude immunodeficient mice (nu/nu), 6–8 weeks old, were purchased from Guangdong Province Medical Animal Center, and feeded and monitored in a specific pathogen-free environmentat Tsinghua University Shenzhen Graduate School. The mice were injected subcutaneously with NSCLC H1975 cells (2.5 × 10^6^) suspended in 100 μL RPMI-1640 medium into the rightflank of each mouse^59^,^61^. Treatments were started when the tumors reached a palpablesize. Mice were randomly divided into three groups (n = 10) and treated with HHT (10 mg/kg), Gefitinib (30 mg/kg) or vehicle control for 3 weeks. Vernier caliper measurements of the longest perpendicular tumor diameters were conducted along with the mice treatment to estimate the tumor volume, using the following formula: 4π/3×(width/2)^2^×(length/2), representing the 3-dimensional volume of an ellipse tumor tissue. Animals were sacrificed when tumors reached to 2 cm or if the mice appeared moribund to prevent unnecessary morbidity to the mice. At the time of the animals’ death, tumors were excised; cells were separatedand lyzed for western blot using anti-STAT3 antibody, anti-pSTAT3, anti-MCL1 and anti-GAPDH antibodies and immunohistochemistry.

### Tumor tissue fluorescent immunohistochemistry

Frozen sections (10 μm) fluorescent immunohistochemistry were prepared as previously described^62^. Briefly, Tumor tissues were surgically excised and cutted into small sections for further fixation with 4% paraformaldehyde for 1 h. The fixed tumor tissue samples were dehydrated in a series of 10%, 20% and 30% sucrose solutions, and then the samples were wrapped into Tissue-Tek O.C.T. compound (Sakura Finetek, USA) and frozen at −80 °C. The samples were frozensectioned into 4- to 10-micron thick sections. After blocking with 3% BSA/0.2% Triton X-100 in PBS for 1 h, sections were incubated with anti-pSTAT3 (Y705) (1:50, Epitomics) antibody at 4 °C overnight. FITC conjugated goat anti-rabbit IgG antibody (1:50, Pierce) were used as the secondary antibody. For observation of cell nucleus, DAPI was used. Sections were observed by Olympus confocal laser scanning microscope with an imaging software (Olympus Fluoview FV-1000, Tokyo, Japan).

### Statistical analysis

All experiments were repeated at least three times and the datawere presented as the mean ± SD unless noted other wise. Differences between data groups were evaluated for significance using Student *t*-test of unpaired data or oneway analysis of variance and Bonferroni post-test. *P* values less than 0.05 indicate statistical significance.

## Additional Information

**How to cite this article**: Cao, W. *et al*. Homoharringtonine induces apoptosis and inhibits STAT3 via IL-6/JAK1/STAT3 signal pathway in Gefitinib-resistant lung cancer cells. *Sci. Rep*. **5**, 8477; doi: 10.1038/srep08477 (2015).

## Supplementary Material

Supplementary Information

## Figures and Tables

**Table 1:** HHT and DTX combination index (CI) values.

A549 cells			H1975 cells		
HHT (nM)	DTX (nM)	CI	HHT (nM)	DTX (nM)	CI
1000	2	0.439	125	2	0.609
2000	8	0.851	250	8	0.715

(A549 and H1975 cells were treated with HHT and DTX combinedly or alone with indicated concentrations for 24 h, the cytotoxicity was analyzed by MTT assay, and the CI values were calculated using CalcuSyn software (Version 2.1).)
