# Supplementary material for: Homoharringtonine induces apoptosis and inhibits STAT3 via IL-6/JAK1/STAT3 signal pathway in Gefitinib-resistant lung cancer cells
Source: Sci Rep. 2015 Jul 13;5:8477. doi: 10.1038/srep08477 (PMC4499885; doi:10.1038/srep08477)
Supplement: Supplementary Information [file srep08477-s1.pdf]

Supplementary information

Homoharringtonine induces apoptosis and inhibits STAT3 via IL-6/JAK1/STAT3 signal pathway in Gefitinib-resistant lung cancer cells

Wei Cao [1,2#], Ying Liu[2,3#], Ran Zhang[1,2], Bo Zhang[4], Teng Wang[2,5], Xianbing Zhu[1,2], Lin Mei[1,2], Hongbo Chen[1,2], Hongling Zhang[1,2],Pinghong Ming[6] and Laiqiang Huang[1,2*]

[1]School of Life Sciences, Tsinghua University, Beijing, 100084, China;

[2]The Shenzhen Key Laboratory of Gene and Antibody Therapy, State Key Laboratory of Health Science and Technology (prep), Ministry-Province Joint Nurturing Base for State Key Lab-Shenzhen Key Laboratory of Chemical Biology, Center for Biotechnology and Biomedicine and Division of Life and Health Sciences, Graduate School at Shenzhen, Tsinghua University, Shenzhen, Guangdong, 518055, China;

[3]School of Basic Medical Sciences, Hubei University of Medicine, Shiyan, 442000, Hubei, China;

[4]National Laboratory of Biomacromolecules, Institute of Biophysics, Chinese Academy of Sciences, Beijing, 100101, China;

[5]The Key Laboratory of Bioorganic Phosphorus Chemistry & Chemical Biology (Ministry of Education), Department of Chemistry, Tsinghua University, Beijing, 100084, China;

[6]Laboratory of Zhuhai People's Hospital, Zhuhai, Guangdong, 519000, China.

***Corresponding author:** The Shenzhen Key Laboratory of Gene and Antibody Therapy, State Key Laboratory of Health Science and Technology (prep), Ministry-Province Joint Nurturing Base for State Key Lab-Shenzhen Key Laboratory of Chemical Biology, Center for Biotechnology and Biomedicine and Division of Life and Health Sciences, Graduate School at Shenzhen, Tsinghua University, Shenzhen, Guangdong, 518055, China. Tel: 86-755-26036012; Fax: 86-755-26036012; E-mail: huanglq403@yahoo.com.

[#] These authors contributed equally to this work.

Supplementary information includes six figures.

Supplementary Figure Legends

Supplementary Figure 1. HHT has no effect on EGFR phosphorylation. Full scan images of immunoblots for main figures.

Supplementary Figure 2. HHT induces apoptosis of NSCLC cells. Full scan images of immunoblots for main figures.

Supplementary Figure 3. HHT supresses the phosphorylation of STAT3. Full scan images of immunoblots for main figures.

Supplementary Figure 4. HHT inhibits IL-6-induced STAT3 phosphorylationin a dose- and time-dependent manner. Full scan images of immunoblots for main figures.

Supplementary Figure 5. HHT exerts synergistic effect combining with docetaxel. Full scan images of immunoblots for main figures.

Supplementary Figure 6. *In vivo* therapeutic efficiency of HHT on mice xenograft bearing human Gefitinib-resistant H1975 cells. Full scan images of immunoblots for main figures.

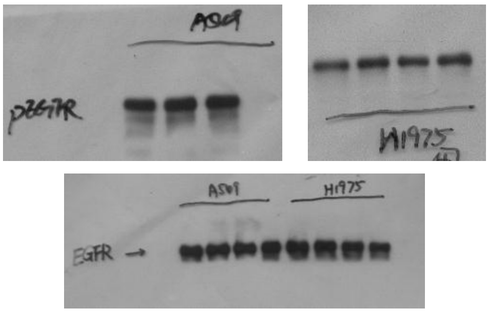

Supplementary Figure 1

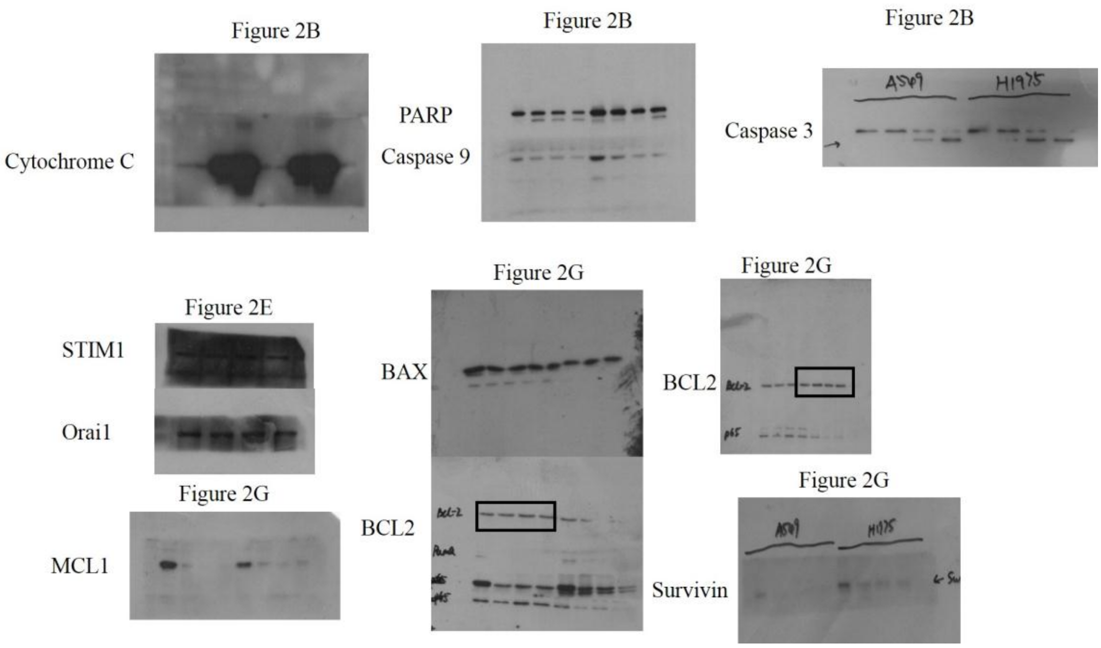

Supplementary Figure 2

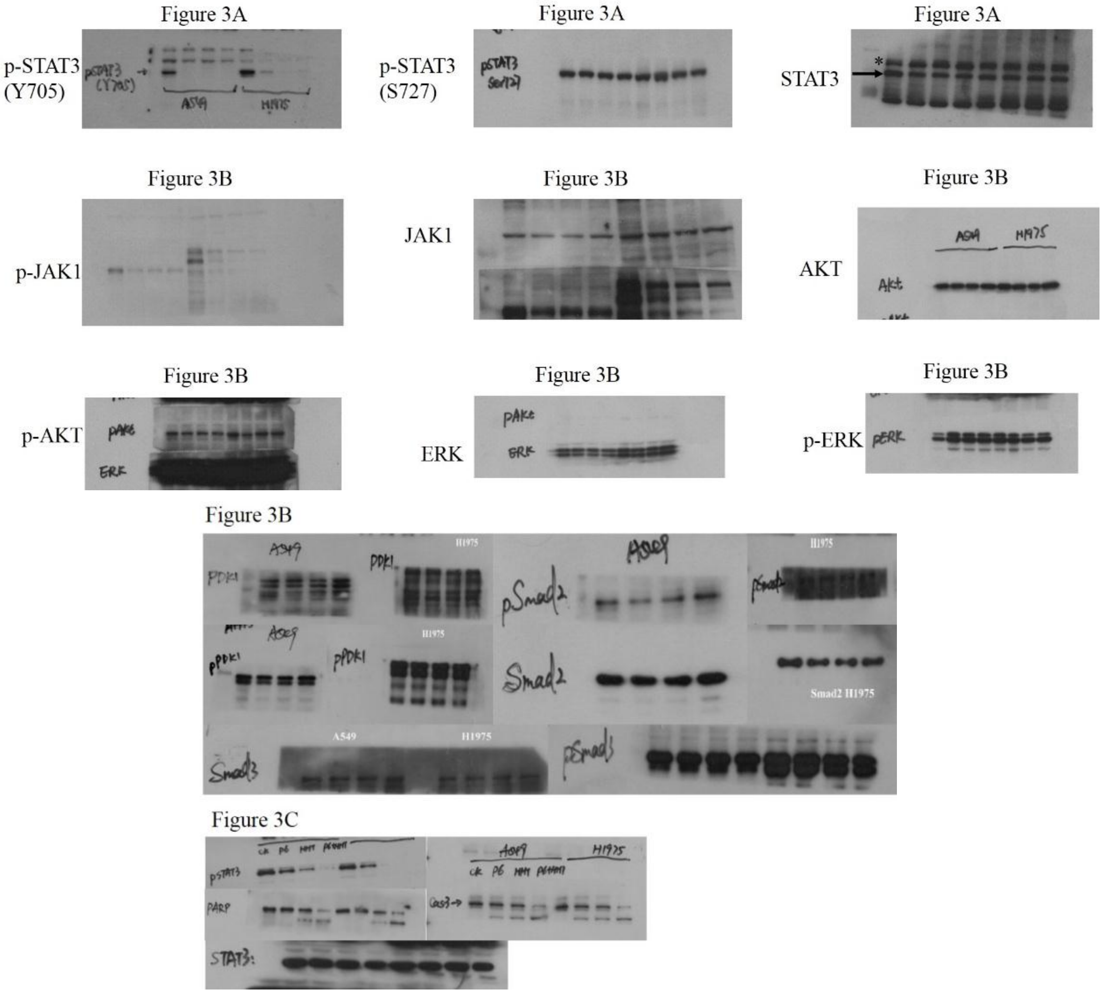

Supplementary Figure 3

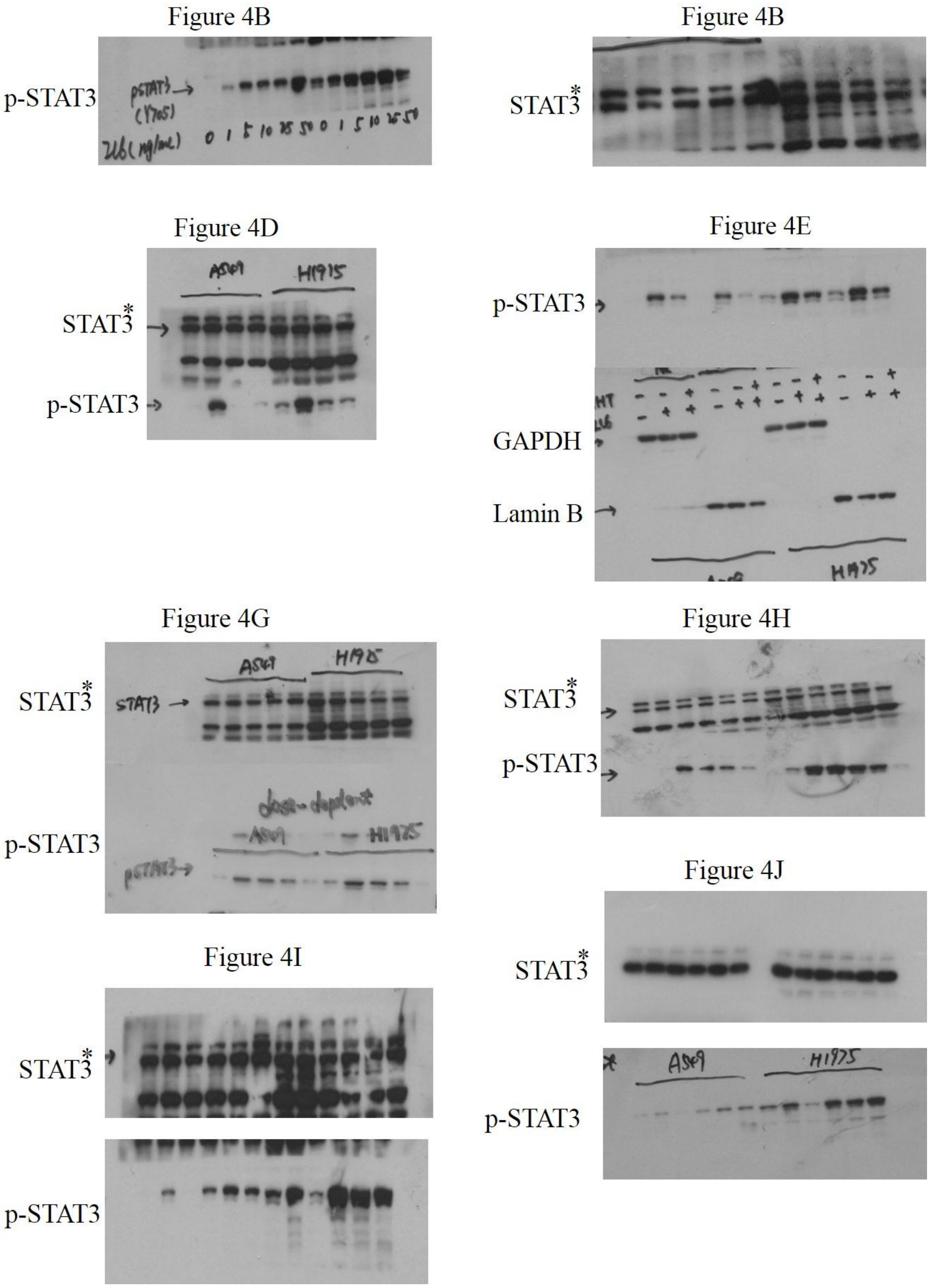

Supplementary Figure 4

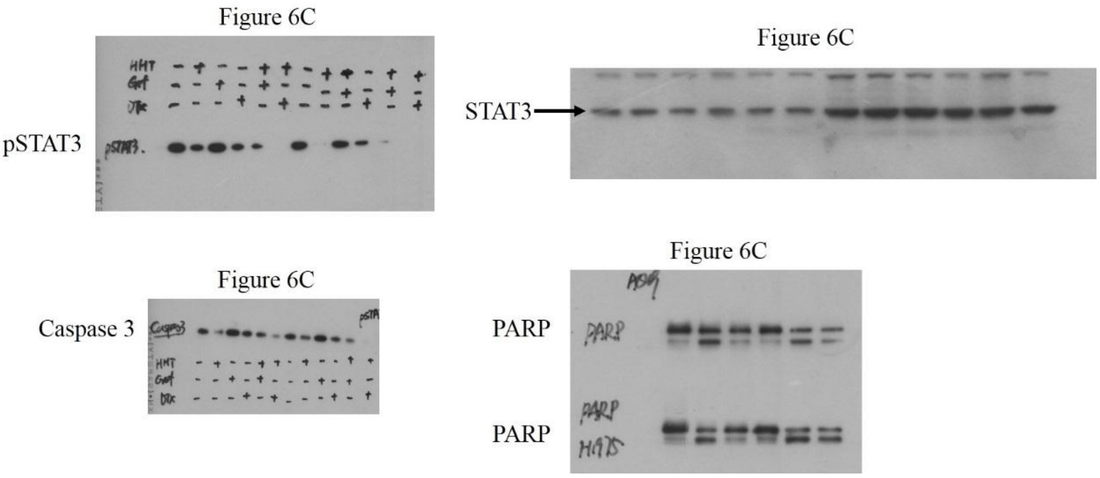

Supplementary Figure 5

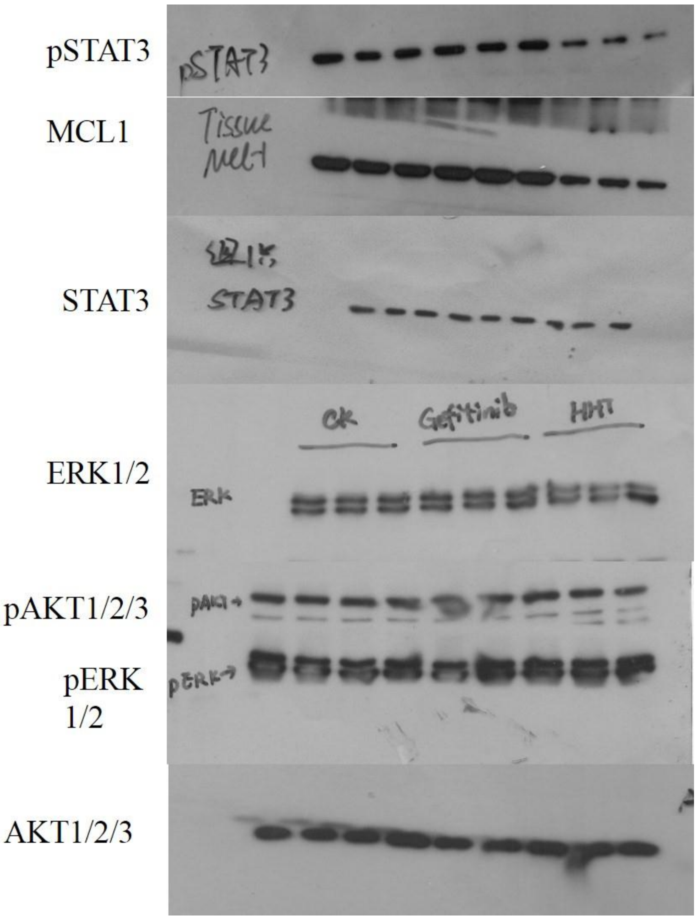

Supplementary Figure 6
